# Posterior Spinal Fixation Using a Patient-Specific 3D-Printed Guide for a Sixth Thoracic Vertebral Fracture With Ankylosis and Severe Bone Sclerosis Due to Spondyloepiphyseal Dysplasia Congenita

**DOI:** 10.7759/cureus.83329

**Published:** 2025-05-01

**Authors:** Taichi Kimura, Satoshi Baba, Mitsumasa Hayashida, Takao Mae

**Affiliations:** 1 Department of Orthopedic Surgery, Saga-Ken Medical Centre Koseikan, Saga, JPN

**Keywords:** ankylosing spinal disorder, patient specific 3d-printed guide, pedicle screw, posterior spinal fixation, vertebral fracture

## Abstract

Vertebral fractures with ankylosing spinal disorder (ASD) are often painful due to the high level of instability and can lead to false joints and delayed paralysis; therefore, surgery is often required. Fixation using percutaneous pedicle screw (PPS) allows for minimally invasive fixation of the spine and is considered useful for ASD cases, but there is a risk of nerve damage during surgery and implant-related complications. Using a patient-specific 3D-printed guide, we were able to perform posterior spinal fixation without intraoperative complications in a patient with vertebral fractures with ASD, which was considered difficult to fix with PPS due to the person's past surgical experience.

## Introduction

Vertebral fractures with ankylosing spinal disorder (ASD) are often reverse chance-type injuries and require firm fixation because of the instability caused by three-column injuries. Because of the lengthening of the lever arm due to intervertebral fusion and the posterior deviation of the screw with the progression of fracture crush with the patient ambulated after surgery, some extensive fixation is considered necessary to prevent the fracture site from collapsing.

The general 2 above-2 below fixation is considered insufficient [1], and 3 above-3 below fixation or insertion of a screw through the vertebral endplate is used [2,3]. However, compared to vertebral fractures of non-ASD, pedicle screws tend to deviate outside more easily, and this is due to the difficulty in confirming the pedicles by X-ray fluoroscopy [4].

In this study, we performed thoracic posterior spinal fixation (PSF) using a patient-specific 3D-printed guide for a sixth thoracic (Th6) vertebral fracture with ASD due to spondyloepiphyseal dysplasia congenita (SEDC) and obtained good results.

## Case presentation

A 53-year-old woman presented to our hospital with back pain after a fall. She has had SEDC, a disease resulting from mutations in the type 2 collagen gene (COL2A1), which leads to short stature, vertebral deformity, and limited joint range of motion. She was short, 120 cm tall, and had contractures in both shoulder, elbow, hip, and knee joints. She had difficulty raising both upper limbs to only around 90 degrees and could not sit up. When she visited the hospital, she was able to walk with crutches as usual and had no lower limb weakness or sensory impairment.

**Figure 1:**
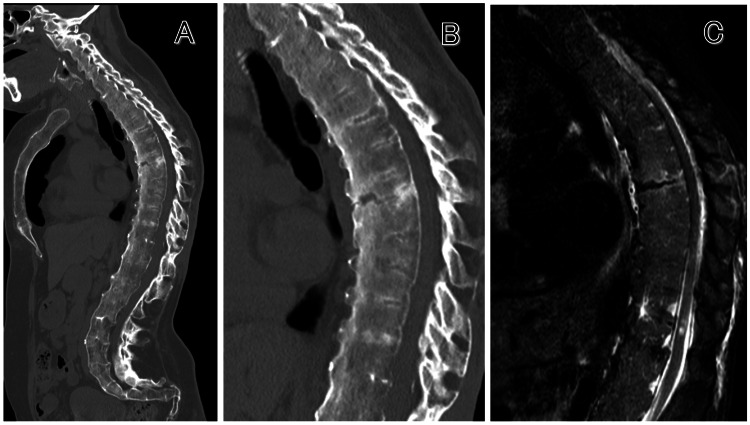
Preoperative images. (A, B) Computed tomography scan showing ankylosis of the entire spine and reverse chance-type sixth thoracic vertebral fracture. (C) Magnetic resonance image showing no obvious damage to the posterior ligament complex.

Computed tomography (CT) and magnetic resonance image (MRI) showed ankylosis of the entire spine and a reverse chance-type Th6 vertebral fracture with anterior vertebral expansion originating from the spinous process (Figure 1), leading to the decision to perform Th3.4.5-Th7.8.9 PSF.

Eight years prior to presentation, the patient had C6 and Th12 vertebral body fractures, for which surgeries（C2.3.4.5-Th2.3.4 PSF and Th9.10.11-L1.2.3 PSF) were performed. At that time, CT-based navigation was used for cervical spine surgery, while X-ray fluoroscopy was used for thoracolumbar spine surgery. At the thoracolumbar level, it was very difficult to identify some pedicles. Also, due to severe osteosclerosis of the cortical bone, normal manual probing was not possible, and thus the probe had to be hammered in. After achieving bone union, the hardware was removed.

**Figure 2:**
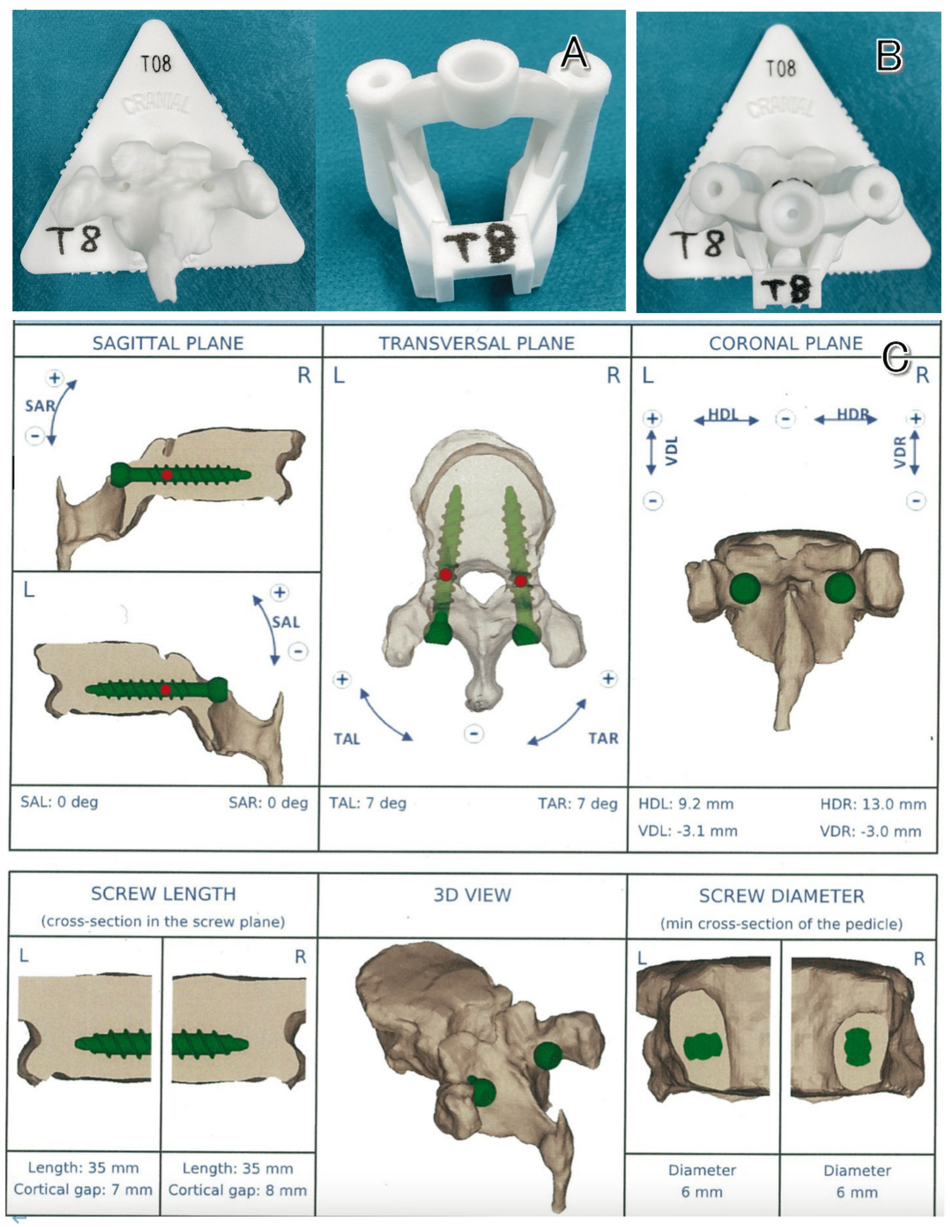
Patient-specific guide and screw planning on the computer. (A) Vertebral body model and guide with drilling holes. (B) Combination of both. (C) Three-dimensional planning of screw insertion angle to prevent the screw from deviating outside the pedicle (Th5).

Therefore, we decided to perform surgery using a patient-specific 3D-printed guide (My Spine®︎, Medacta SA International, Castel San Pietro, Switzerland) that does not require X-ray fluoroscopy and allows probing through a drilling procedure, thereby reducing mechanical stress on the thoracic spinal cord. This guide is based on computer planning of proper screw insertion points and trajectories for each vertebral body extracted from preoperative CT scans. Based on this planning, a guide with a vertebral body model and drilling holes for screw insertion can be created using a 3D printer (Figure 2).

We created the guides from Th3 to Th9 vertebrae, and surgery was performed. During the planning process, we ensured that the screw entry points were set so that it would not shift medially or laterally to facilitate rod fixation. We also determined the insertion angles and lengths of the screws within the pedicle in three CT planes (axial, sagittal, and coronal). Additionally, on the left side, the screw length was not too close to the anterior vertebral wall of the vertebral body to avoid arterial injury.

**Figure 3:**
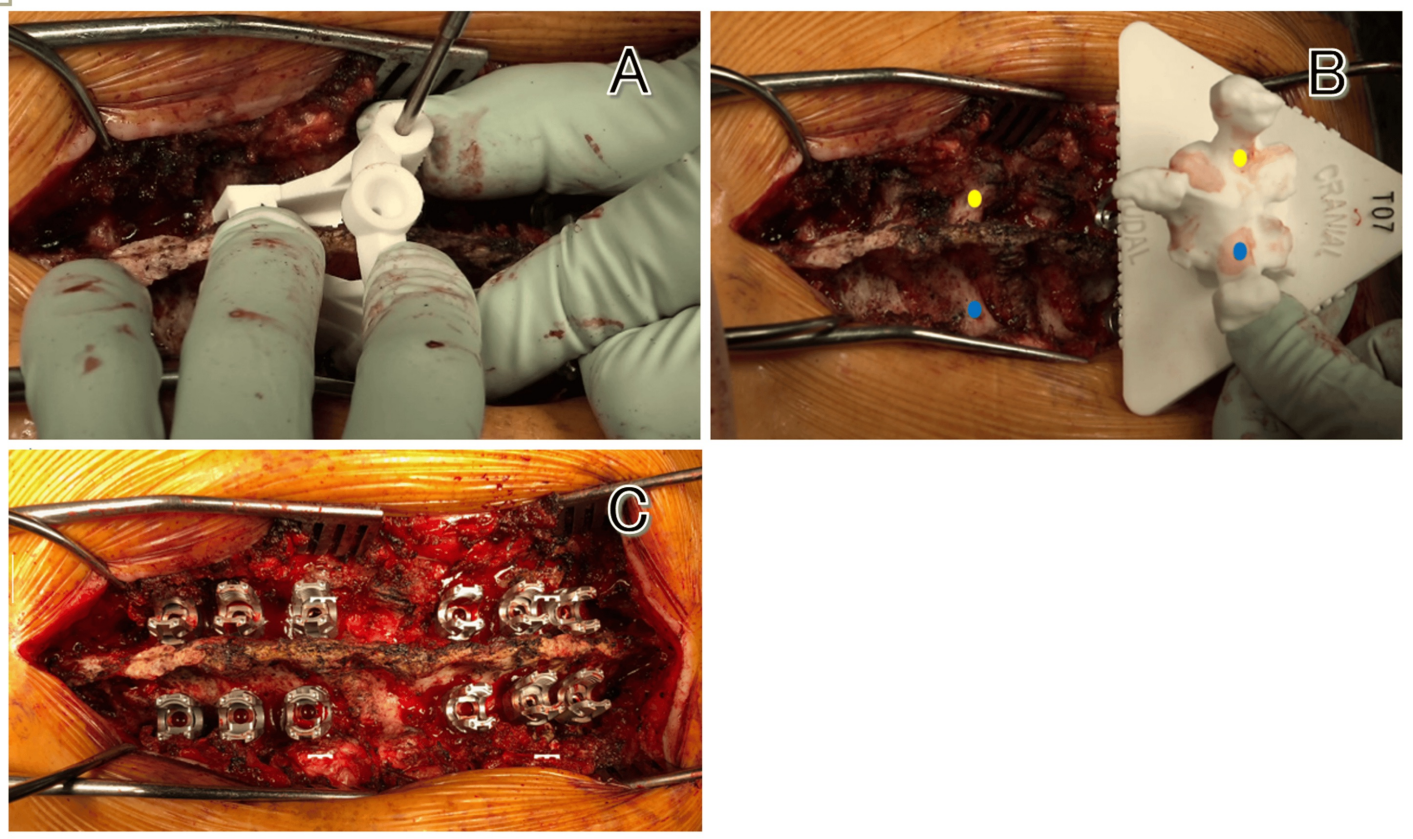
Intraoperative image. (A) The guide is fitted to the vertebral arch and drilled through the hole. (B) The screw insertion point is the same as the drill hole in the vertebral body model (Th7). (C) All screws are inserted using the guide. The right is the head side and the left is the foot side.

The surgical position was set to the Concorde position, with consideration of thoracic kyphosis to prevent the fracture site from expanding. A midline longitudinal incision was made from the Th3 to Th9, and the soft tissues attached to the spinous processes and laminae were adequately dissected and removed to expose the contact points for the guide. The screw holes from the previous hardware removal at the Th3 and Th4 vertebrae were checked to confirm the levels. Starting from the cranial side, the patient-specific 3D-printed guides were fitted, and after drilling and tapping were performed to create pilot holes, the planned sizes of pedicle screws were inserted (Figure 3).

As expected, severe bone sclerosis was observed at the screw insertion site. After all the screws were inserted, titanium rods bent in a kyphotic direction were placed on both sides and craniocaudal fastening was performed.

**Figure 4:**
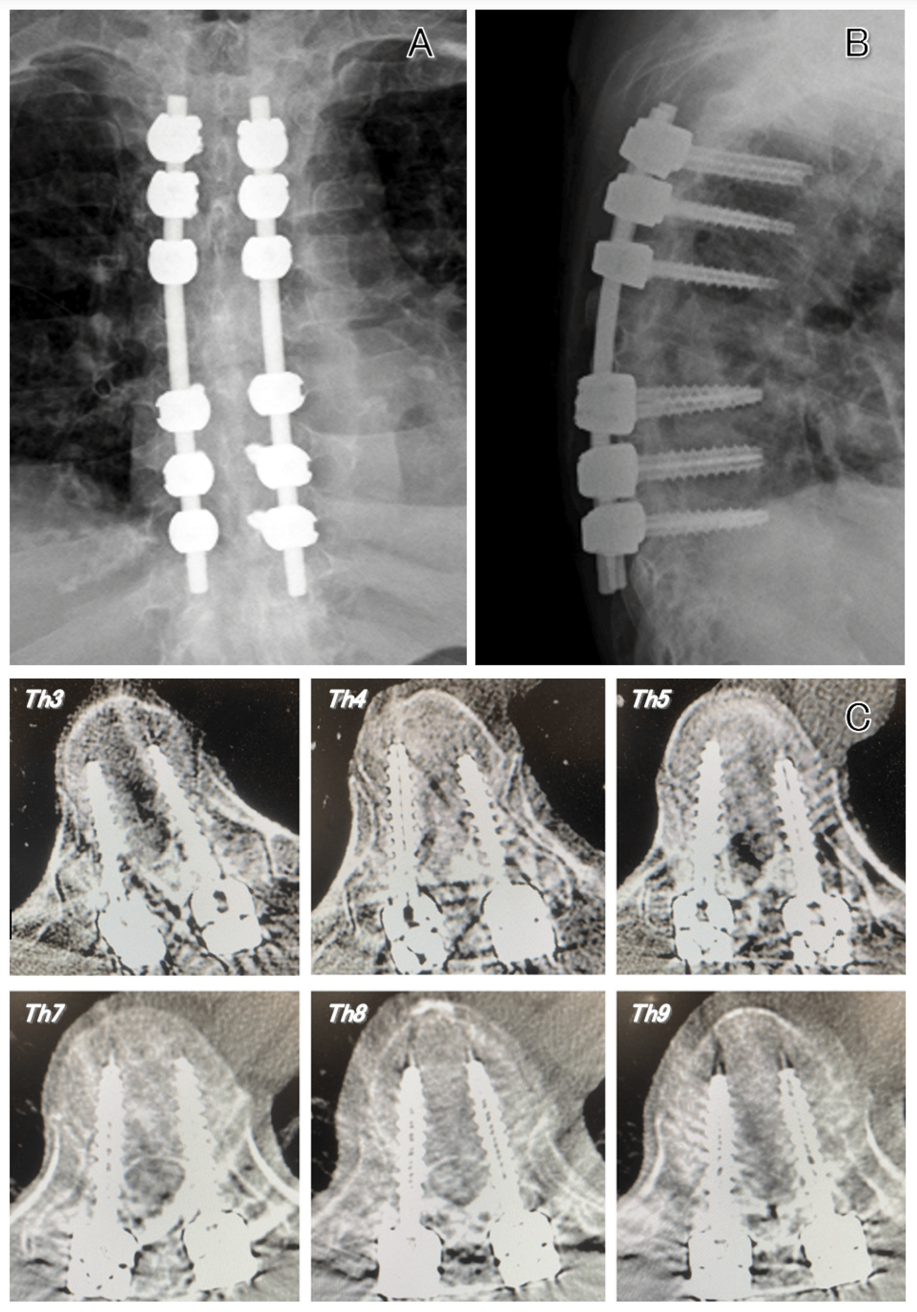
Postoperative images. (A, B) X-ray fluoroscopy shows that the rods are almost parallel on both sides, and the screws are long enough to be inserted. (C) Computed tomography scan shows none of the screws deviated outside the pedicles.

The patient began walking training with crutches four days after surgery and returned to work as a teacher at a school for children with disabilities four months after surgery. The screws were inserted according to the preoperative planning, and no screws were deviated outside the pedicles (Figure 4).

As of two years after surgery, the patient has not experienced any late complications.

## Discussion

Recently, posterior spinal fixation using patient-specific 3D-printed guide has become widespread and is used for various procedures such as spinal degenerative diseases, deformities, and trauma.

Many studies have reported that the device allows for highly accurate and safe insertion of pedicle screws [5-7]. It has also been reported that the surgery time was reduced by approximately 30% compared to the freehand technique under X-ray fluoroscopy [8], leading to a decrease in radiation exposure [9]. Additionally, another advantage is that it does not require investment in special equipment such as navigation devices. In cases of vertebral fractures with ASD, screws may be inserted through the endplate using the SEPS/DEPS (single or double endplate penetrating screw) technique [3]. When using this method, the screw must be inserted at a stronger oblique angle than usual. In case of severe osteosclerosis of the cortical bone, it is difficult to create the screw insertion point using normal probing. We believe that drilling using a patient-specific 3D-printed guide makes it easy to insert screws without having to worry about the probe tip slipping.

However, it requires two weeks to create the device, making it difficult to use in emergency surgeries. Furthermore, the guide placement may be unstable if there is severe degeneration, if there is little bone remaining after laminectomy, or if the guide contact area is damaged intraoperatively [5]. Another disadvantage is the additional cost involved in creating the device (200 USD per vertebra, plus approximately 100 USD for each additional vertebra) [10].

There are not many reports of its use in trauma, likely due to the preparation time required; however, if lower limb paralysis or bladder/rectal dysfunction does not occur, it is possible to wait until surgery, making it safer than performing emergency surgery with uncertain methods.

In our case, a four-week waiting period was required, but the patient did not suffer any disadvantages such as spinal cord injury during that time. During surgery, there was little degeneration of the spinous processes, vertebral arches, or transverse processes, making it possible to place stable guides on each vertebra. Furthermore, the insertion of the pedicle screws was smooth and accurate due to the drilling procedure, and the surgery was performed safely.

## Conclusions

Patient-specific 3D-printed guide takes time to prepare, which requires a waiting period before surgery; however, by creating a guide based on careful planning, surgery can be performed in a simple and safe manner. We believe that in cases of vertebral fractures with ASD, where the fracture site is highly unstable and it is sometimes difficult to identify the pedicle by X-ray fluoroscopy, there is a particular possibility that the risk of complications such as spinal cord injury may be significantly reduced. Further clinical studies are required to confirm the risk of complications and the amount of X-ray radiation exposure associated with the use of this guide.

## References

[1] Okada E, Shiono Y, Nishida M (2019). Spinal fractures in diffuse idiopathic skeletal hyperostosis: advantages of percutaneous pedicle screw fixation. J Orthop Surg (Hong Kong).

[2] Werner BC, Samartzis D, Shen FH (2016). Spinal fractures in patients with ankylosing spondylitis: etiology, diagnosis, and management. J Am Acad Orthop Surg.

[3] Takeuchi T, Hosogane N, Yamagishi K, Satomi K, Matsukawa K, Ichimura S (2020). Results of using a novel percutaneous pedicle screw technique for patients with diffuse idiopathic skeletal hyperostosis-the single or double endplates penetrating screw (SEPS/DEPS) technique. Spine Surg Relat Res.

[4] Sasagawa T, Marubayashi N, Hashimoto N (2017). Accuracy of percutaneous pedicle screw placement: a comparison study between ankylosing spinal disorder and non-ankylosing spinal disorder. Clin Orthop Surg.

[5] Cool J, van Schuppen J, de Boer MA, van Royen BJ (2021). Accuracy assessment of pedicle screw insertion with patient specific 3D‑printed guides through superimpose CT-analysis in thoracolumbar spinal deformity surgery. Eur Spine J.

[6] Lamartina C, Cecchinato R, Fekete Z, Lipari A, Fiechter M, Berjano P (2015). Pedicle screw placement accuracy in thoracic and lumbar spinal surgery with a patient-matched targeting guide: a cadaveric study. Eur Spine J.

[7] Putzier M, Strube P, Cecchinato R, Lamartina C, Hoff EK (2017). A new navigational tool for pedicle screw placement in patients with severe scoliosis: a pilot study to prove feasibility, accuracy, and identify operative challenges. Clin Spine Surg.

[8] Kaya I, Cingöz İD, Şahin MC, Atar M, Ozyoruk S, Sayin M, Yuceer N (2021). Are 3D printing templates an advantage in upper thoracic pedicle screw fixation?. Cureus.

[9] Farshad M, Betz M, Farshad-Amacker NA, Moser M (2017). Accuracy of patient-specific template-guided vs. free-hand fluoroscopically controlled pedicle screw placement in the thoracic and lumbar spine: a randomized cadaveric study. Eur Spine J.

[10] Kaito T, Matsukawa K, Abe Y, Fiechter M, Zhu X, Fantigrossi A (2018). Cortical pedicle screw placement in lumbar spinal surgery with a patient-matched targeting guide: a cadaveric study. J Orthop Sci.

